# Castleman Disease—Still More Questions than Answers: A Case Report and Review of the Literature

**DOI:** 10.3390/jcm14082799

**Published:** 2025-04-18

**Authors:** Mariusz Sikora, Nel Dąbrowska-Leonik, Piotr Buda, Beata Wolska-Kuśnierz, Karina Jahnz-Różyk, Małgorzata Pac, Ewa Więsik-Szewczyk

**Affiliations:** 1 Department of Internal Medicine, Pneumonology, Allergology, Clinical Immunology and Rare Diseases, Military Institute of Medicine-National Research Institute, 04-141 Warsaw, Poland; kjrozyk@wim.mil.pl; 2 Department of Immunology, Children’s Memorial Health Institute, 04-730 Warsaw, Poland; n.dabrowska-leonik@ipczd.pl (N.D.-L.); b.wolska-kusnierz@ipczd.pl (B.W.-K.); m.pac@ipczd.pl (M.P.); 3 Department of Pediatrics, Nutrition and Metabolic Disorders, Children’s Memorial Health Institute, 04-730 Warsaw, Poland; p.buda@ipczd.pl

**Keywords:** case report, Castleman disease, fever of unknown origin, lymphadenopathy, tocilizumab, transitional care

## Abstract

**Background:** Idiopathic multicentric Castleman disease (iMCD) is a rare lymphoproliferative disorder with diverse clinical presentations, often posing significant diagnostic challenges. **Methods:** We report the case of a 20-year-old woman who first presented with fever of unknown origin (FUO) at the age of 14, followed by the development of abdominal lymphadenopathy. We conducted a comprehensive review of her clinical course, diagnostic workup, treatment response, and outcomes. Additionally, we performed a literature review of CD focusing on pathophysiology, classification, diagnostic approaches, and treatment strategies. **Results:** Extensive investigations performed in the meantime excluded infectious and autoimmune causes. Histopathological analysis of the excised lymph nodes ruled out malignancy and confirmed idiopathic multicentric Castleman disease. Treatment with tocilizumab, an IL-6 receptor antagonist, resulted in rapid resolution of clinical symptoms, normalization of inflammatory markers, and sustained remission. With the final diagnosis established and treatment initiated, she was transitioned at the age of 18 from a pediatric immunology to an adult clinical immunology center. **Conclusions:** The presented case highlights the importance of considering iMCD in the differential diagnosis of FUO, especially in adolescents, and the efficacy of targeted therapies in managing this challenging disease. A multidisciplinary approach involving clinical, laboratory, imaging, and histopathological evaluation is essential for accurate diagnosis. IL-6 pathway inhibition represents an effective targeted therapy for iMCD, capable of inducing sustained remission in this challenging disease.

## 1. Introduction

Castleman disease (CD) was first described in 1956 by Dr. Benjamin Castleman, who presented several case reports of a single enlarged lymph node, most commonly in the mediastinum, which exhibited distinct vascularity and hyalinization [1]. Currently, the term CD describes a group of rare and heterogeneous polyclonal lymphoproliferative disorders with various subtypes characterized by multicentric follicular hyperplasia and abundant plasma cell proliferation [2]. Based on the clinical presentation, number of affected lymph node regions, and disease course, CD is generally classified into two main types: unicentric and multicentric. Unicentric CD (UCD) involves a solitary affected lymph node and usually follows a benign clinical course with no or mild symptoms, whereas multicentric CD (MCD), which manifests with extensive lymph node involvement and the presence of systemic symptoms, carries a worse prognosis [3].

The exact pathogenesis of CD still remains not fully elucidated, but it is thought to involve immune system dysregulation and abnormal cytokine production [2]. In particular, interleukin-6 (IL-6) has been identified as a key mediator in the pathogenesis of the disease. Overproduction of IL-6 results in hyperactivation of B and plasma cells, contributing to the development of the characteristic lymph node enlargement and systemic inflammatory response [4]. The disease can occur with similar frequency in both men and women at any age, however it is rare in children and adolescents [3]. Most cases of UCD can be treated with surgical excision of the affected lymph nodes, whereas the management of MCD is more challenging and may require immunosuppressive therapies [5]. The prognosis and mortality in MCD patients are highly variable, with five-year overall survival rates of 50–77% [6]. The main causes of mortality in cases of iMCD include progressive multi-organ failure due to uncontrolled systemic inflammation, infectious complications from immunosuppressive therapy, thrombotic events, and the development of secondary malignancies, particularly lymphomas. However, use of the targeted therapies has markedly improved patient outcomes [7].

Nearly 70 years after its initial description, CD continues to be a complex and intriguing disease with numerous unanswered questions. We report the case of an adolescent with prolonged fever of unknown origin (FUO) who underwent extensive diagnostic evaluation and multiple hospital admissions before being diagnosed with CD. Despite its rarity, accurate documentation of CD cases remains crucial to raise awareness and deepen our understanding of this condition. Therefore, in this case report, we presented the clinical details and management of a patient diagnosed with CD, along with a literature review of the disease.

## 2. Case Description

This case describes a 20-year-old woman who presented her first symptoms at the age of 14 and after a broad diagnostic process was transitioned at the age of 18 from pediatric immunology center to adult immunology center with a final diagnosis of idiopathic MCD (iMCD) and established treatment. The patient provided written informed consent for the access to her clinical data and publication of this case report.

### 2.1. Initial Presentation (Age 14)

The patient’s medical history began with fever, fatigue, and weight loss of approximately 7 kg over two months. She experienced daily episodes of temperature increases up to 40 °C that lasted from two days up to three weeks. Initially, these spikes occurred every 1–2 weeks, but after six months began to appear every day. The patient denied coughing, breathing difficulties, joint pain, skin rashes, or night sweats. There was no history of recent travel, contact with sick individuals, or illicit drug use. Her medical and family history was unremarkable and she was fully vaccinated.

### 2.2. Early Diagnostic Workup and Management (Age 14–15)

The patient received courses of oral antibiotics from her general practitioner including amoxicillin with clavulanic acid, azithromycin, clarithromycin, and ciprofloxacin. As there was no clinical response, she was admitted several times through the year for further evaluation to general pediatric, pediatric hematology, and rheumatology departments.

**Figure 1:**
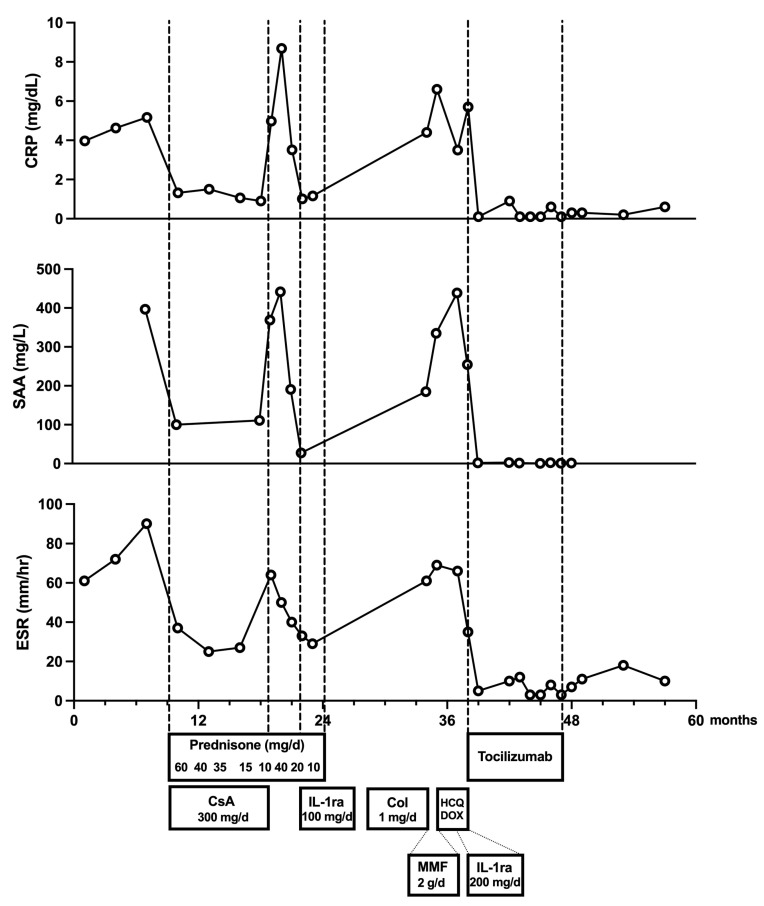
Changes in inflammatory markers and corresponding treatment interventions over time. The x-axis represents the number of months since the onset of the patient’s symptoms. Abbreviations: CRP—C-reactive protein, SAA—serum amyloid A, ESR—erythrocyte sedimentation rate, CsA—ciclosporin A, IL-1ra—interleukin-1 receptor antagonist (anakinra), Col—colchicine, MMF—mycophenolate mofetil, HCQ—hydroxychloroquine, DOX—doxycycline.

During these initial hospitalizations, physical exams were normal, without lymphadenopathy. Standard laboratory tests showed a hypochromic microcytic anemia and elevated inflammatory markers (shown in Figure 1). Serum protein electrophoresis revealed polyclonal hypergammaglobulinemia with hypoalbuminemia. Extensive investigations for infectious causes, including cultures, serologies, and interferon-gamma release assay, were negative. Antinuclear antibody titer was marginally positive (1:160), while complement levels were normal. Imaging with chest X-ray, abdominal ultrasound, and echocardiogram showed no abnormalities. Bone marrow evaluation demonstrated hypercellularity with slightly suppressed erythropoiesis and an active granulopoiesis system, along with an expanded reticuloendothelial compartment containing numerous plasma cells.

### 2.3. Tertiary Center Evaluation and First Surgical Intervention (Age 15)

After ruling out infections, hematological malignancies, and systemic connective tissue disease as the cause of FUO, the patient was admitted to a tertiary referral hospital (Children’s Memorial Health Institute, Warsaw, Poland). Blood analysis confirmed elevated inflammatory parameters, including an erythrocyte sedimentation rate of 90 mm/h (reference range < 20 mm/h), a high-sensitivity C-reactive protein of 5.17 mg/L (reference < 0.5), serum amyloid A of 507 mg/L (reference < 6.4), and procalcitonin of 2.6 mg/dL (reference < 0.1). 

One of the subsequent ultrasound examinations revealed for the first time enlarged lymph nodes in the paraaortic region. An MRI confirmed a cluster of enlarged lymph nodes measuring 80 × 35 × 23 mm in the paraaortic area, with signs of restricted water diffusion and discreet contrast enhancement. The report also mentioned enlarged lymph nodes near the aortocaval area and in the hepatic hilum. A partial laparoscopic resection of the lymph nodes cluster was performed. Histological analysis revealed enlarged lymph nodes with hyalinized connective tissue, alongside extravasations, hemorrhages and inflammatory infiltrates consisting of histiocytes, polyclonal plasma cells, and granulocytes—suggestive of an inflammatory pseudotumor.

### 2.4. Initial Treatment Attempts (Age 15–16)

Treatment with glucocorticoids was introduced, initially pulses with methylprednisolone followed by prednisone at a dose of 1 mg/kg b.w. with ciclosporin 300 mg daily (5 mg/kg b.w.). The fever subsided and the laboratory inflammatory parameters normalized.

### 2.5. Disease Progression and Second Surgery (Age 16)

As the prednisone dose was gradually reduced over the following months to 10 mg/day, the fever recurred and inflammatory markers increased again, despite continued treatment with ciclosporin. A follow-up MRI scan showed enlargement of the masses in the cluster of lymph nodes with increased contrast enhancement. Subsequently, one year after the first surgery, a laparotomy was performed with complete resection of the remaining cluster of enlarged lymph nodes. Histopathological examination of this specimen confirmed the previous findings of an inflammatory pseudotumor, with no evidence of malignancy.

### 2.6. Further Treatment Attempts (Age 16–17)

Corticosteroid therapy continued after hospitalization, but fever recurred when the dose was tapered. A subsequent attempt was made to include anakinra (100 mg/day) into the treatment regimen, followed by colchicine (1 mg/day), and later mycophenolate mofetil (2 g/day). However, this did not improve the clinical and laboratory markers of inflammation. Furthermore, a control MRI examination revealed numerous lymph nodes in the hepatic hilum, enlarged retroperitoneal lymph nodes, and subcapsular lymph nodes in the spleen.

### 2.7. Genetic Testing and Dose Escalation (Age 17)

Along with the suspicion of an underlying autoinflammatory disease, a whole-exome sequencing (WES) analysis was performed. It revealed a heterozygous variation in the ZC3H12C gene at position NM_033390.2: c.1819C>T, resulting in the amino acid change p.Pro607Ser (variant of uncertain significance). This genetic variant was inherited from the father, while the copy from the mother is inactive due to genomic imprinting mechanisms. No functional validation was performed. An attempt was made to reapply anakinra, increasing its dose to 200 mg/day. However, only a partial improvement was achieved, characterized by a decrease in the frequency and/or intensity of evening fever spikes, but not their complete cessation, and a reduction in inflammatory parameters, but not their normalization. Following lack of complete remission, an FDG-PET/CT was performed as part of further diagnostics, revealing mild-to-moderate increased tracer accumulation in reactive lymph nodes of the neck, chest, and abdominal cavity.

### 2.8. Final Diagnosis and Effective Treatment (Age 17–18)

The histology samples from both the first (laparoscopic resection) and second (laparotomy) interventions were reevaluated, showing Castleman disease-like changes classified as hypervascular type. The IL-6 reached serum concentration of a level of 45.5 pg/mL (reference range < 7.0 pg/mL). The patient was diagnosed with iMCD and started treatment with tocilizumab at a dose of 8 mg/kg every 2–3 weeks.

### 2.9. Transition to Adult Care, Treatment Outcome and Follow-Up (Age 18–20)

This therapy resulted in a rapid improvement in clinical symptoms, normalization of laboratory inflammatory markers, resolution of lymphadenopathy, and steroids discontinuation. All therapeutic interventions were well tolerated throughout the treatment course, and no adverse events or treatment intolerance were reported during the follow-up period. Tocilizumab maintenance therapy was stopped after nine months of treatment. Since then, regular check-ups have shown that the patient remains asymptomatic and her condition is stable without any sign of recurrence within a two-year follow-up period. The clinical course and diagnostic-therapeutic interventions are summarized in Table 1.

## 3. Discussion

This illustrative case emphasizes the potential difficulties in the diagnosis and treatment dilemmas of CD in adolescence. We reported the case of a 20-year-old patient who initially presented with FUO at the age of 14, later developing lymphadenopathy that directed the diagnosis toward iMCD, which, after histopathological confirmation, enabled the initiation of targeted treatment.

### 3.1. Epidemiology of Castleman Disease

Idiopathic multicentric Castleman disease can affect individuals of all ages, but it typically presents in adults with a median age of diagnosis around 50 years [8]. In contrast, the epidemiology of CD in adolescents remains poorly understood due to its rarity and the limited number of reported cases in this age group [9,10]. Thus, our case makes a unique contribution to the existing literature, emphasizing the need for dedicated research on CD within adolescent populations.

### 3.2. Castleman Disease in Differential Diagnosis of FUO

Fever of unknown origin is defined as well-documented fever with a body temperature > 38.3 °C lasting for at least three weeks without any apparent source after one week of investigation in the hospital [11]. It remains a challenging issue in clinical practice and accounts for approximately 3% of hospital admissions, significantly impacting healthcare systems [12]. There are over 200 reported causes of FUO in children, including various infectious, inflammatory, rheumatologic, and malignant conditions, which could potentially explain the clinical presentation observed in this case [11,13].

Infections account for 16–55% of FUO cases and common causative factors include abscesses, endocarditis, tuberculosis, and complicated urinary tract infections [14,15]. However, the presence of lymphadenopathy and an abdominal mass limits the list of potential diagnoses in the presented case. Given the results of all laboratory microbiological tests and the lack of a response to courses of empirical antibiotic treatment, it was unlikely that the patient’s symptoms were caused by an infection.

Cancer accounts for around 2–25% of FUO cases. Neoplasms most frequently associated with FUO include renal cell carcinoma, lymphomas, hepatocellular, and ovarian cancer, as well as atrial myxoma [16,17]. The presence of an abdominal mass, lymphadenopathy, splenomegaly, and general symptoms raised suspicion for lymphoma. Nonetheless, histopathological examination of the mass combined with low FDG uptake in PET/CT argued against the diagnosis of cancer.

Immune-mediated disorders, including systemic juvenile idiopathic arthritis, connective tissue diseases, sarcoidosis, and IgG4-related disease, represent another significant group of FUO causes (comprising between 5% and 22%) [13,18]. However, these are unlikely diagnoses in this case, based on the imaging, biopsy, and laboratory findings. While the initial idea of an autoinflammatory disease seemed plausible, the absence of confirmation in a genetic test and insufficient response to high doses of an IL-1 receptor antagonist required further validation of the diagnosis.

Later, the patient met the diagnostic criteria for iMCD based on international, evidence-based consensus [19]. Our patient had multicentric lymphadenopathy with defined histopathology, presented with 6 out of 11 minor criteria: elevated CRP, anemia, hypoalbuminemia, polyclonal hypergammaglobulinemia, constitutional symptoms, and splenomegaly. Additionally, diseases that mimic iMCD were ruled out. Although nondiagnostic, the elevated interleukin-6 level and the low FDG uptake also supported the diagnosis of iMCD.

### 3.3. Classification of Castleman Disease

As mentioned, Castleman’s disease was initially described by Dr. Benjamin Castleman in 1954 [1]. Since then, various types of CD have been defined based on clinical, pathological, and virological characteristics. Unicentric CD is a localized disease affecting a single lymph node [20]. MCD is systemic and progressive condition characterized by lymphadenopathy in multiple nodes [3]. MCD can be further subclassified according to its etiology into human herpes virus-8 (HHV-8)-associated MCD (HHV8-MCD), polyneuropathy, organomegaly, endocrinopathy, monoclonal gammopathy and skin changes (POEMS)-associated MCD (POEMS-MCD), and idiopathic MCD, which refers to patients without HHV-8 infection or POEMS syndrome manifestations. The latter subtype can be further classified based on clinical manifestation into iMCD-TAFRO (thrombocytopenia, ascites, reticulin fibrosis, renal dysfunction, organomegaly) or subtype with less severe clinical course—iMCD-NOS (not otherwise specified) [3,5].

### 3.4. Clinical Manifestations of Castleman Disease

Castleman disease can manifest with a variety of clinical signs and symptoms. UCD typically presents with solitary (unifocal) lymph node enlargement, commonly in the mediastinum, axillary, or inguinal regions and the abdominal cavity [2,20]. Additionally, there have been reports of extranodal occurrences of CD in the pancreas [21], lacrimal glands [22], and intramuscularly [23]. While constitutional symptoms are uncommon in UCD, some patients may experience systemic manifestations [24]. MCD, while presenting with prominent lymphadenopathy, usually causes additional nonspecific, alarming, systematic symptoms like weight loss, night sweats with fever, and anasarca edema, leading patients to seek medical care. Other findings include organomegaly, especially hepato/splenomegaly, renal dysfunction, and respiratory complications. One of the clinical challenges associated with iMCD is its often non-specific initial presentation. Several case reports highlight FUO as the initial manifestation of iMCD, particularly in TAFRO syndrome, often preceding the development of more characteristic features such as lymphadenopathy [25,26,27]. The clinical patterns of fever in iMCD vary widely, ranging from intermittent to continuous, and may persist for weeks or months, as observed in our case report [28,29]. Nishimura et al. reported that while fever was present in almost all patients with TAFRO syndrome, it occurred less frequently (28.6%) in iMCD-NOS cases, where it was typically intermittent [27]. These observations highlight the heterogeneity of fever presentation in CD and the need to include iMCD in the differential diagnosis of FUO. However, despite its diagnostic relevance, data on fever in iMCD are primarily derived from case reports and letters to the Editor, limiting detailed characterization and making the true prevalence and clinical patterns of fever difficult to quantify.

The severity of MCD ranges from few clinical and laboratory abnormalities to multi-organ failure. In severe cases, such as POEMS or TAFRO syndrome, MCD can be life-threatening [6]. However, some patients may exhibit mild or asymptomatic clinical courses despite the involvement of multiple lymph nodes [30].

### 3.5. Diagnosis of Castleman Disease

Diagnosing CD presents a significant clinical challenge due to the wide range of clinical manifestations presented above. Numerous diseases can mimic CD and according to the guidelines, these must be excluded before diagnosing iMCD [19]. Moreover, iMCD can co-occur with various autoimmune (myasthenia gravis, psoriasis) or autoinflammatory (familial mediterranean fever, Schnitzler’s syndrome) conditions [31,32].

#### 3.5.1. Imaging Tests

Imaging findings are often nonspecific in cases of CD [33]. Contrast-enhanced CT (CECT) scans are commonly used in CD patients to identify and characterize lymph nodes by size, shape, and contrast enhancement pattern. However, CT imaging cannot sensitively detect the involvement of normal-sized lymph nodes nor distinguish between reactive hyperplasia and pathological enlargement. Due to limited scanning field, CT scans cannot promptly evaluate the system involvement of CD [33,34]. Whole-body 18F-Fluorodeoxyglucose positron emission tomography-CT (F-FDG PET-CT) imaging shows increased but variable standardized uptake value (SUV) of CD-involved lesions [35]. The standardized uptake value (SUVmax) in CD-involved lesions typically ranges from mild to moderate (2.5–5.0) in most cases, consistent with the metabolic activity typical of inflammatory processes, rather than exceeding the thresholds commonly associated with malignancy (SUVmax > 10). However, the glucose metabolism of CD is associated with gender, clinical classification, and histopathological subtype, as well as lesion size and volume, making it difficult to differentiate CD from malignant disorders [36]. Some reports suggest that combined PET/CT and CECT scans in CD cases may offer better results than PET/CT or CECT imaging alone [36]. Imaging studies played a crucial role in diagnosing Castleman disease in our patient. The ability to perform repeated abdominal ultrasound examinations allowed for the detection of enlarged lymph nodes in the abdominal cavity, which appeared later during the course of the disease and remained clinically asymptomatic. MRI provided a more detailed assessment of lymph node clusters, particularly in complex anatomical regions such as the retroperitoneum and mediastinum. Consequently, follow-up MRI scans were utilized in our patient to monitor disease activity during treatment. PET/CT imaging was especially valuable in differentiating CD from malignant conditions, such as lymphoma.

#### 3.5.2. Histopathology

Even though imaging techniques were crucial in detecting lymphadenopathy in this case of iMCD, particularly when FUO was the initial presentation, final diagnosis required histopathological confirmation. An excisional lymph node biopsy followed by histological examination remains the gold standard for definitive diagnosis of CD [19]. In addition to the diverse clinical presentation mentioned earlier, CD also exhibits a variety of histologic features. There are three main patterns observed in CD-affected lymph nodes: hyaline vascular (HV-CD) and plasma cell infiltration pattern (PC-CD), which represent two ends of a spectrum of histological variance rather than distinct entities, along with the mixed pattern at the center of this spectrum [3,37,38]. In HV pathology, there is an enlargement of lymphoid follicular structure with thickening in the blood vessels and glass-like changes in later stages. The follicle is surrounded by multilayer of circular lymphocytes forming the characteristic “onion skin” structure. In contrast, PC-CD is characterized by proliferating plasma cells at all levels between follicles. Reticular lymph nodes are large with few transparent vessels and generally no typical onion skin structure is present. The mixed pattern has characteristics of both HV-CD and the plasmacytic variant. Many researchers previously believed that HV pathology occurs only in UCD, but HV morphology recently has been found in iMCD patients, leading to defining them specifically as hypervascular histopathological subtype [2,38,39].

#### 3.5.3. Genetic Tests

Genetic testing is not routinely performed in the diagnosis of CD [19]. However, in certain cases—especially when atypical symptoms are present or there is suspicion of coexisting autoinflammatory monogenic disease—genetic testing can be helpful. Whole-exome sequencing analysis has identified potential gene variants that play a role in the development and prognosis of CD [40,41]. These studies emphasize that genes affecting chromatin organization and abnormalities in methylation (such as SETD1A, ASH1L, KMT2E, and DNMT3A) are more prevalent in iMCD [42,43], while abnormalities in interleukin signaling pathways (PDGFRB, FGFR3, NF1, PIM1, PTPN6, IL6ST) occur more frequently in UCD [40,43]. Genetic mutations within the mitogen-activated protein kinase (MAPK) pathway were found in both UCD and iMCD [43]. Alterations in five genes (NCOA4, DARS2, MTCL1, RABPE1, and DNAH11) are associated with unfavorable clinical outcomes in iMCD patients [41]. Among these, NCOA4 (nuclear receptor coactivator 4), also known as ARA70 (androgen receptor-associated protein 70) is of particular interest. Abnormal expression and function of NCOA4 have been linked to carcinogenesis. In several cases of iMCD, a specific point mutation c.781>T has been observed, resulting in the encoding of p.Leu 261Phe (L261F) which affects a highly conserved region of the gene [41]. Based on structural modeling, it is predicted that the L261F mutation leads to instability in the protein structure, potentially altering its conformation and phenotype. Notably, no other malignancies harboring an NCOA4 mutation encoding L261F have been identified in the literature, suggesting that NCOA4 L261F mutations may be highly specific to iMCD [41]. The WES analysis on our patient did not identify a clear genetic cause for the observed symptoms. The detected gene variant ZC3H12C: c.1819C>T, p.Pro607Ser is classified as a variant of uncertain significance. In humans, single nucleotide polymorphisms (SNPs) in ZC3H12C have been recognized as a risk factor for psoriasis susceptibility in several genome-wide association studies [44]. ZC3H12C (also known as Regnase-3 or monocyte chemotactic protein-induced protein 3; MCPIP-3) is member of the ZC3H12 family and acts as an RNase essential for maintaining immune homeostasis [45]. Studies using human umbilical vein endothelial cells (HUVEC) have demonstrated that ZC3H12C suppresses proinflammatory activity by inhibiting the NF-κB signaling pathway and blocking the transcription and expression of TNF-induced chemokines and adhesion molecules [46]. Plasmacytoid dendritic cells deficient in ZC3H12C secrete significantly higher amounts of IL-6 [47], while ZC3H12C-deficient mice developed hypertrophic lymph nodes along with a higher proportion of immature B-cells and innate immune cells via IFN signaling in myeloid cells [48]. However, we emphasize that these experimental findings cannot be directly extrapolated to interpret the clinical significance of the variant found in our patient. The role of ZC3H12C variant (p.Pro607Ser) in iMCD remains speculative due to the absence of functional validation. Further studies are warranted to explore its biological significance.

### 3.6. Pathophysiology of Castleman Disease

The exact cause of iMCD is still not fully elucidated; however, it is thought that the symptoms result from the release of various cytokines, including interleukins and vascular endothelial growth factor (VEGF), with IL-6 playing a crucial role in the development of CD [49]. IL-6 has the ability to increase VEGF secretion, which contributes significantly to angiogenesis and vascular permeability. It also affects B cell proliferation and differentiation into antibody-producing cells, leading to follicular hyperplasia and lymph node enlargement [50]. The proposed mechanisms for stimulating cytokine production include viral hypothesis, paraneoplastic syndrome with ectopic cytokine secretion hypothesis, or autoimmune background [51].

### 3.7. Treatment of Castleman Disease

According to the International Treatment Consensus for iMCD, management depends on the severity of the disease. For non-severe cases, therapy targeting IL-6 is considered first-line treatment option: siltuximab, an IL-6 neutralizing chimeric monoclonal antibody, and tocilizumab, a humanized monoclonal antibody that binds to the IL-6 receptor [52]. Siltuximab is currently the only FDA and EMA-approved treatment for patients with iMCD who are negative for HHV8 and HIV.

Due to the lack of reimbursement for siltuximab at the time, we opted to treat the patient with tocilizumab. The availability of siltuximab and tocilizumab varies among countries. As there have been no head-to-head trials comparing their efficacy so far, the choice between them currently depends more on reimbursement policies and access within each country’s healthcare system [52].

Tocilizumab (8 mg/kg b.w. i.v. every two weeks) is recommended as an alternative first-line treatment when siltuximab is unavailable [52]. In our case, this therapy rapidly alleviated symptoms and led to long-lasting remission. This medication has similar safety and effectiveness profiles compared to siltuximab, although these findings have not been confirmed in a randomized controlled trial [53]. A small, open-label, nonrandomized, prospective single-arm study involving 35 iMCD patients showed that 86% of those who continued the treatment for at least five years benefit from tocilizumab [54]. However, the objective response criteria were not shown. The drug has been approved for the treatment of iMCD in Japan and extensively documented in literature [53]. The duration of targeted therapy in iMCD can vary widely, ranging from several months to years, depending on the patient’s response and the stability of remission [55,56]. Treatment decisions should be personalized, made collaboratively between the physician and patient, and guided by continuous monitoring of disease activity [52]. Although no definitive guidelines specify the exact duration of therapy, in our case, tocilizumab was administered at a dose of 8 mg/kg body weight every two weeks until full control of clinical symptoms and normalization of inflammatory markers (CRP, SAA) was achieved. Once stable remission was achieved, maintenance therapy with tocilizumab was continued every three weeks up to nine months. After discontinuing therapy, we implemented close monitoring through regular clinical assessments, imaging studies, and laboratory tests to promptly detect any signs of relapse.

If IL-6 targeted antibodies are not available or if patients remain refractory to therapy with siltuximab or tocilizumab, the alternative treatment should consist of rituximab (375 mg/m^2^ i.v., short-term therapy: 4–8 doses), which is an anti-CD20 monoclonal antibody. Corticosteroids can be implemented at any stage of the therapeutic regimen. Other treatment options for patients who do not respond to the above include thalidomide, lenalidomide, bortezomib, ciclosporin, sirolimus, or anakinra [52]. In our patients, only a high dose of anakinra (200 mg/day) showed a partial response. There are limited case reports and studies evaluating anakinra in iMCD, often in patients with refractory disease [57] or those with overlapping autoinflammatory features [32]. In such cases, anakinra has sometimes been used off-label, but results are inconsistent. Some patients experience significant and rapid improvement in symptoms and laboratory abnormalities [57], while others show partial [58] or no response [59].

### 3.8. The Importance of Multidisciplinary Approach in Diagnosing Castleman Disease

The diagnosis and management of iMCD require a multidisciplinary team approach due to the complexity and rarity of the condition. Effective diagnosis relies on collaboration among clinicians, including hematologists, immunologists, and rheumatologists, together with pathologists and radiologists, to integrate clinical, laboratory, imaging, and histopathological data. As demonstrated by Pelliccia et al. (2023) in a study of cases with Castleman-like histological features, a structured histopathological review combined with clonality testing significantly improves diagnostic accuracy [60]. The involvement of specialists from multiple disciplines is also important for differentiating iMCD from mimicking conditions, such as autoimmune diseases and IgG4-related disorders. Given the complexity of iMCD and the evolving landscape of its treatment, input from various specialists is essential for optimizing targeted therapies. In our case, a collaborative approach involving specialists from pediatrics, rheumatology, hematology, radiology, pathology, and clinical immunology was crucial for diagnosis and management. This emphasizes that iMCD care, as recommended by the Castleman Disease Collaborative Network (CDCN) guidelines, requires systematic integration of diverse expertise to improve patient outcomes [60].

## 4. Conclusions

Idiopathic multicentric Castleman disease is a rare disease with a highly diverse clinical presentation that can mimic many different conditions, including infections, lymphomas, autoimmune diseases, and autoinflammatory syndromes. The histopathological examination of the removed lymph node remains the gold standard for an accurate diagnosis. This case report aims to highlight the importance of considering iMCD in the differential diagnosis of FUO and lymphadenopathy. Early initiation of targeted therapy is crucial for achieving remission and reducing the risk of organ damage. Inhibition of IL-6, with siltuximab or tocilizumab, has transformed the management of iMCD, offering effective disease control and improving patient outcomes. In our case, tocilizumab led to rapid symptom resolution, normalization of inflammatory markers, and sustained remission, underscoring the role of biologic therapies in iMCD treatment. Given the variability in treatment response and disease course, individualized therapeutic strategies and long-term follow-up are essential to optimize patient care.

## Figures and Tables

**Table 1:** Step-by-Step Timeline of the Patient’s Clinical Course and Therapeutic Intervention.

Time	Clinical Course and Interventions
Age 14	Fever of unknown origin, fatigue, and significant weight loss
Age 14–15	Early diagnostic workup including multiple hospital admissions Laboratory investigations: elevated inflammatory markers, anemia, hypergammaglobulinemia Imaging (chest X-ray, ultrasound, echocardiogram) without significant findings Infectious disease workup (negative) Multiple antibiotic courses—no clinical response
Age 15	Tertiary center evaluation at Children’s Memorial Health Institute Abdominal ultrasound and MRI revealing enlarged lymph nodes in the paraaortic region First surgical intervention: partial laparoscopic resection of lymph node cluster Histopathological analysis suggesting an inflammatory pseudotumor
Age 15–16	Treatment with glucocorticoids (methylprednisolone pulses, then prednisone) combined with ciclosporin—initial improvement followed by recurrence of fever and inflammatory markers upon steroid tapering
Age 16	Disease progression noted on follow-up MRI: enlargement of lymph node clusters Second surgery (laparotomy) with complete resection of remaining lymph node masses Histopathology confirmed inflammatory pseudotumor (no malignancy)
Age 16–17	Further treatment attempts: introduction of anakinra (100 mg/day), followed by colchicine and mycophenolate mofetil—no improvement in clinical or laboratory parameters
Age 17	Genetic testing (WES) revealed heterozygous ZC3H12C gene variant (NM_033390.2: c.1819C>T, p.Pro607Ser)—variant of uncertain significance Dose escalation of anakinra to 200 mg/day—partial improvement (reduced evening fever spikes, partial reduction of inflammatory markers)
Age 17–18	FDG-PET/CT—increased tracer uptake in reactive lymph nodes Reevaluation of biopsy samples from both surgical interventions—Castleman disease-like changes (hypervascular type) Final diagnosis of idiopathic multicentric Castleman disease Initiation of targeted therapy with tocilizumab (8 mg/kg every 2–3 weeks)—rapid clinical improvement, normalization of inflammatory markers
Age 18–20	Transition from pediatric to adult clinical immunology care Resolution of lymphadenopathy, steroid discontinuation Tocilizumab discontinued after nine months of treatment Patient remains asymptomatic with no disease recurrence during two-year follow-up period

## Data Availability

The original data presented in the study are included in the article, further inquiries can be directed to the corresponding author.

## Abbreviations

The following abbreviations are used in this manuscript:

CD	Castleman disease
UCD	unicentric Castleman disease
MCD	multicentric Castleman disease
IL	interleukin
FUO	Fever of unknown origin
iMCD	idiopathic multicentric Castleman disease
MRI	magnetic resonance imaging
WES	whole-exome sequencing
FDG-PET/CT	fluorodeoxyglucose-positron emission tomography/computed tomography
CRP	C-reactive protein
HHV-8	human herpes virus-8
POEMS	polyneuropathy, organomegaly, endocrinopathy, monoclonal gammopathy, and skin changes
TAFRO	thrombocytopenia, ascites, reticulin fibrosis, renal dysfunction, organomegaly
NOS	not otherwise specified
CECT	contrast-enhanced computed tomography
SUV	standardized uptake value
HV-CD	hyaline vascular Castleman disease
PC-CD	plasma cell infiltration pattern Castleman disease
HUVEC	human umbilical vein endothelial cells
TNF	tumor necrosis factor
VEGF	vascular endothelial growth factor
FDA	Food and Drug Administration
EMA	European Medicines Agency
HIV	human immunodeficiency virus
SAA	serum amyloid A

## References

[1] Castleman B., Towne V.W. (1954). Case records of the Massachusetts General Hospital: Case No. 40231. N. Engl. J. Med..

[2] Carbone A., Borok M., Damania B., Gloghini A., Polizzotto M.N., Jayanthan R.K., Fajgenbaum D.C., Bower M. (2021). Castleman disease. Nat. Rev. Dis. Primers.

[3] Nishimura M.F., Nishimura Y., Nishikori A., Yoshino T., Sato Y. (2022). Historical and pathological overview of Castleman disease. J. Clin. Exp. Hematop..

[4] Kishimoto T., Kang S. (2022). IL-6 Revisited: From Rheumatoid Arthritis to CAR T Cell Therapy and COVID-19. Annu. Rev. Immunol..

[5] Dispenzieri A., Fajgenbaum D.C. (2020). Overview of Castleman disease. Blood.

[6] Fajgenbaum D.C., Pierson S.K., Kanhai K., Bagg A., Alapat D., Lim M.S., Lechowicz M.J., Srkalovic G., Uldrick T.S., van Rhee F. (2022). The disease course of Castleman disease patients with fatal outcomes in the ACCELERATE registry. Br. J. Haematol..

[7] Pierson S.K., Lim M.S., Srkalovic G., Brandstadter J.D., Sarmiento Bustamante M., Shyamsundar S., Mango N., Lavery C., Austin B., Alapat D. (2023). Treatment consistent with idiopathic multicentric Castleman disease guidelines is associated with improved outcomes. Blood Adv..

[8] Robinson D., Reynolds M., Casper C., Dispenzieri A., Vermeulen J., Payne K., Schramm J., Ristow K., Desrosiers M.P., Yeomans K. (2014). Clinical epidemiology and treatment patterns of patients with multicentric Castleman disease: Results from two US treatment centres. Br. J. Haematol..

[9] Hu C., Zou Y., Pan J., Yang J., Yang T., Tan T., Li J. (2020). Analysis of Clinical Characteristics, Pathological Changes and Changes of Interleukin-6 (IL-6) and C-Reactive Protein (CRP) in Children with Castleman’s Disease. Med. Sci. Monit..

[10] Parez N., Bader-Meunier B., Roy C.C., Dommergues J.P. (1999). Paediatric Castleman disease: Report of seven cases and review of the literature. Eur. J. Pediatr..

[11] Trapani S., Fiordelisi A., Stinco M., Resti M. (2023). Update on Fever of Unknown Origin in Children: Focus on Etiologies and Clinical Approach. Children.

[12] Cunha B.A. (2007). Fever of unknown origin: Clinical overview of classic and current concepts. Infect. Dis. Clin. N. Am..

[13] Haidar G., Singh N. (2022). Fever of Unknown Origin. N. Engl. J. Med..

[14] Wright W.F., Yenokyan G., Simner P.J., Carroll K.C., Auwaerter P.G. (2022). Geographic Variation of Infectious Disease Diagnoses Among Patients With Fever of Unknown Origin: A Systematic Review and Meta-analysis. Open Forum Infect. Dis..

[15] Nowroozizadeh B., Haghighi Mehmandari N., Gallegos N., Perez-Rosendahl M., Lu D. (2017). Q Fever Presented as a Large Retroperitoneal Pseudotumoral Mass. Case Rep. Pathol..

[16] Foggo V., Cavenagh J. (2015). Malignant causes of fever of unknown origin. Clin. Med..

[17] Sogaard K.K., Farkas D.K., Leisner M.Z., Schmidt S.A.J., Lash T.L., Sorensen H.T. (2022). Fever of Unknown Origin and Incidence of Cancer. Clin. Infect. Dis..

[18] Milchert M., Makowska J., Brzezinska O., Brzosko M., Wiesik-Szewczyk E. (2019). Monogenic autoinflammatory diseases in adults—A challenge to rheumatologic practice at the onset of the Polish national programme of interleukin 1 inhibitor treatment. Reumatologia.

[19] Fajgenbaum D.C., Uldrick T.S., Bagg A., Frank D., Wu D., Srkalovic G., Simpson D., Liu A.Y., Menke D., Chandrakasan S. (2017). International, evidence-based consensus diagnostic criteria for HHV-8-negative/idiopathic multicentric Castleman disease. Blood.

[20] Molacek J., Treska V., Skalicky T., Vodicka J., Ferda J., Ferdova E., Baxa J., Mach C., Jungova A., Michal M. (2023). Unicentric form of Castleman s disease, pitfalls of diagnosis and surgical treatment. Front. Oncol..

[21] Kaniewska M., Wasielica-Berger J., Wereszczyńska-Siemiątkowska U., Augustynowicz A., Cepowicz D., Kędra B., Dąbrowski A. (2007). Case report Castleman’s disease imitating pancreatic tumour—Diagnostic difficulties. Gastroenterol. Rev./Prz. Gastroenterol..

[22] Li D., Tang D., Sun F. (2020). Clinical Analysis of Castleman’s Disease of the Lacrimal Gland. J. Ophthalmol..

[23] Eward W.C., DeWitt S.B., Brigman B.E., Kontogeorgakos V., Lagoo A.S. (2014). Extranodal Castleman disease of the extremities: A case report and review of the literature. Skelet. Radiol..

[24] Muhammad T., Alkheder A., Mazloum A., Almooay A., Naziha L., Shaheen M. (2024). Unicentric Castleman disease: A case report of an atypical presentation and successful management. Int. J. Surg. Case Rep..

[25] Zambrana Garcia J.L., Torres Serrano F., Jansen Chaparro S., Lopez Rubio F., Jimenez-Pereperez J.A., Perez Jimenez F. (1996). Fever of unknown origin as initial manifestation of Castleman’s disease: Apropos of 2 cases. An. Med. Interna.

[26] Roca B., Torres V. (2009). Castleman’s disease presenting as fever of unknown origin: Diagnostic value of fluorodeoxyglucose-positron emission tomography/computed tomography. Am. J. Med. Sci..

[27] Nishimura Y., Hanayama Y., Fujii N., Kondo E., Otsuka F. (2020). Comparison of the clinical characteristics of TAFRO syndrome and idiopathic multicentric Castleman disease in general internal medicine: A 6-year retrospective study. Intern. Med. J..

[28] Bustamante M.S., Pierson S.K., Ren Y., Bagg A., Brandstadter J.D., Srkalovic G., Mango N., Alapat D., Lechowicz M.J., Li H. (2024). Longitudinal, natural history study reveals the disease burden of idiopathic multicentric Castleman disease. Haematologica.

[29] Lust H., Gong S., Remiker A., Rossoff J. (2021). Idiopathic multicentric Castleman disease with TAFRO clinical subtype responsive to IL-6/JAK inhibition: A pediatric case series. Pediatr. Blood Cancer.

[30] Oksenhendler E., Boutboul D., Fajgenbaum D., Mirouse A., Fieschi C., Malphettes M., Vercellino L., Meignin V., Gerard L., Galicier L. (2018). The full spectrum of Castleman disease: 273 patients studied over 20 years. Br. J. Haematol..

[31] Gonzalez Garcia A., Fernandez-Martin J., Robles Marhuenda A. (2023). Idiopathic multicentric Castleman disease and associated autoimmune and autoinflammatory conditions: Practical guidance for diagnosis. Rheumatology.

[32] Soudet S., Fajgenbaum D., Delattre C., Forestier A., Hachulla E., Hatron P.Y., Launay D., Terriou L. (2018). Schnitzler syndrome co-occurring with idiopathic multicentric Castleman disease that responds to anti-IL-1 therapy: A case report and clue to pathophysiology. Curr. Res. Transl. Med..

[33] Din F., Mellor F., Millard T., Pace E., Khan N., Attygalle A.D., Cunningham D., Zafar S., Sharma B. (2022). Radiology of Castleman disease: The pivotal role of imaging in diagnosis, staging, and response assessment of this rare entity. Clin. Radiol..

[34] Pitot M.A., Tahboub Amawi A.D., Alexander L.F., LeGout J.D., Walkoff L., Navin P.J., Kawashima A., Wood A.J., Dispenzieri A., Venkatesh S.K. (2023). Imaging of Castleman Disease. Radiographics.

[35] Jiang Y., Hou G., Zhu Z., Huo L., Li F., Cheng W. (2021). 18F-FDG PET/CT imaging features of patients with multicentric Castleman disease. Nucl. Med. Commun..

[36] He L., Chen Y., Tan X., Sun X., Zhang Q., Luo H., Jiang L. (2023). (18)F-FDG PET/CT and contrast-enhanced CT in the diagnosis of Castleman disease. Jpn. J. Radiol..

[37] Fajgenbaum D.C., Wu D., Goodman A., Wong R., Chadburn A., Nasta S., Srkalovic G., Mukherjee S., Leitch H., Jayanthan R. (2020). Insufficient evidence exists to use histopathologic subtype to guide treatment of idiopathic multicentric Castleman disease. Am. J. Hematol..

[38] Wu D., Lim M.S., Jaffe E.S. (2018). Pathology of Castleman Disease. Hematol. Oncol. Clin. N. Am..

[39] Liu X.R., Tian M. (2022). Glucocorticoids combined with tofacitinib in the treatment of Castleman’s disease: A case report. World J. Clin. Cases.

[40] Wang S., Wang R., Shang P., Zhu X., Chen X., Zhang G., Wang M. (2024). Whole-Exome Sequencing Reveals the Genomic Profile and IL6ST Variants as a Prognostic Biomarker of Paraneoplastic Pemphigus-Associated Unicentric Castleman Disease. J. Investig. Dermatol..

[41] You L., Lin Q., Zhao J., Shi F., Young K.H., Qian W. (2020). Whole-exome sequencing identifies novel somatic alterations associated with outcomes in idiopathic multicentric Castleman disease. Br. J. Haematol..

[42] Nagy A., Bhaduri A., Shahmarvand N., Shahryari J., Zehnder J.L., Warnke R.A., Mughal T., Ali S., Ohgami R.S. (2018). Next-generation sequencing of idiopathic multicentric and unicentric Castleman disease and follicular dendritic cell sarcomas. Blood Adv..

[43] Butzmann A., Kumar J., Sridhar K., Gollapudi S., Ohgami R.S. (2021). A Review of Genetic Abnormalities in Unicentric and Multicentric Castleman Disease. Biology.

[44] Tsoi L.C., Spain S.L., Knight J., Ellinghaus E., Stuart P.E., Capon F., Ding J., Li Y., Tejasvi T., Gudjonsson J.E. (2012). Identification of 15 new psoriasis susceptibility loci highlights the role of innate immunity. Nat. Genet..

[45] Fischer M., Weinberger T., Schulz C. (2020). The immunomodulatory role of Regnase family RNA-binding proteins. RNA Biol..

[46] Liu L., Zhou Z., Huang S., Guo Y., Fan Y., Zhang J., Zhang J., Fu M., Chen Y.E. (2013). Zc3h12c inhibits vascular inflammation by repressing NF-kappaB activation and pro-inflammatory gene expression in endothelial cells. Biochem. J..

[47] Liu B., Huang J., Ashraf A., Rahaman O., Lou J., Wang L., Cai P., Wen J., Anwaar S., Liu X. (2021). The RNase MCPIP3 promotes skin inflammation by orchestrating myeloid cytokine response. Nat. Commun..

[48] Clayer E., Frank D., Anderton H., Zhang S., Kueh A., Heim V., Nutt S.L., Chopin M., Bouillet P. (2022). ZC3H12C expression in dendritic cells is necessary to prevent lymphadenopathy of skin-draining lymph nodes. Immunol. Cell Biol..

[49] Fajgenbaum D.C., Shilling D. (2018). Castleman Disease Pathogenesis. Hematol. Oncol. Clin. N. Am..

[50] Hirano T. (2021). IL-6 in inflammation, autoimmunity and cancer. Int. Immunol..

[51] Ostrowska B., Romejko-Jarosińska J., Domańska-Czyż K., Walewski J. (2021). Idiopathic multicentric Castleman disease: Pathogenesis, clinical presentation and recommendations for treatment based on the Castleman Disease Collaborative Network (CDCN). Acta Haematol. Pol..

[52] van Rhee F., Voorhees P., Dispenzieri A., Fossa A., Srkalovic G., Ide M., Munshi N., Schey S., Streetly M., Pierson S.K. (2018). International, evidence-based consensus treatment guidelines for idiopathic multicentric Castleman disease. Blood.

[53] Rehman M.E.U., Chattaraj A., Neupane K., Rafae A., Saeed S., Basit J., Ibrahim A., Khouri J., Mukherjee S., Anwer F. (2022). Efficacy and safety of regimens used for the treatment of multicentric Castleman disease: A systematic review. Eur. J. Haematol..

[54] Kapriniotis K., Lampridis S., Mitsos S., Patrini D., Lawrence D.R., Panagiotopoulos N. (2018). Biologic Agents in the Treatment of Multicentric Castleman Disease. Turk. Thorac. J..

[55] Rossini B., Cecchi N., Clemente F., De Paolis M.R., Hohaus S., Innao V., Lucignano M., Massaiu R., Palumbo G., Rigolin G.M. (2024). Real-practice management and treatment of idiopathic multicentric Castleman disease with siltuximab: A collection of clinical experiences. Drugs Context.

[56] Lang E., Sande B., Brodkin S., van Rhee F. (2022). Idiopathic multicentric Castleman disease treated with siltuximab for 15 years: A case report. Ther. Adv. Hematol..

[57] El-Osta H., Janku F., Kurzrock R. (2010). Successful treatment of Castleman’s disease with interleukin-1 receptor antagonist (Anakinra). Mol. Cancer Ther..

[58] Galeotti C., Tran T.A., Franchi-Abella S., Fabre M., Pariente D., Kone-Paut I. (2008). IL-1RA agonist (anakinra) in the treatment of multifocal castleman disease: Case report. J. Pediatr. Hematol. Oncol..

[59] Borocco C., Ballot-Schmit C., Ackermann O., Aladjidi N., Delaleu J., Giacobbi-Milet V., Jannier S., Jeziorski E., Maurier F., Perel Y. (2020). The French paediatric cohort of Castleman disease: A retrospective report of 23 patients. Orphanet J. Rare Dis..

[60] Pelliccia S., Rogges E., Cardoni A., Lopez G., Conte E., Faccini A.L., De Vito R., Girardi K., Bianchi A., Annibali O. (2024). The application of a multidisciplinary approach in the diagnosis of Castleman disease and Castleman-like lymphadenopathies: A 20-year retrospective analysis of clinical and pathological features. Br. J. Haematol..

